# A Unique Case of IGF-2 Induced Hypoglycemia Associated with Hepatocellular Carcinoma

**DOI:** 10.1155/2019/4601484

**Published:** 2019-10-13

**Authors:** Prachi Rana, Brian Kim

**Affiliations:** ^1^Department of Internal Medicine, Los Angeles County Hospital/University of Southern California, USA; ^2^Department of Gastroenterology and Liver Diseases, Los Angeles County Hospital/University of Southern California, USA

## Abstract

Tumor-induced hypoglycemia (TIH) associated with hepatocellular carcinoma (HCC) results from tumor production of insulin-like growth factor 2 (IGF-2), resulting in recurrent hypoglycemia. Serum levels of insulin-like growth factor 1 (IGF-1), insulin-like growth factor 2 (IGF-2), or pro-insulin-like growth factor 2 (pro-IGF-2) are required for the diagnosis. However, due to the limited availability of testing in many laboratories, this condition is often overlooked. As this condition is often overlooked, prompt recognition and implementation of tumor directed therapies and/or systemic therapies can help mitigate the consequences of hypoglycemia. We present a 38-year-old man with advanced multifocal HCC who presented with abdominal pain and was found to have recurrent symptomatic hypoglycemia. Evaluation revealed elevated levels of IGF-2, suggesting a diagnosis of HCC with production of IGF-2.

## 1. Introduction

Tumor-induced hypoglycemia (TIH) is a rare cause of hypoglycemia and is most commonly associated with tumor hyperinsulinism. However, TIH has been recently linked to nonislet-cell tumor hypoglycemia (NICTH) due to the production of insulin-like growth factor 1 (IGF-1), insulin-like growth factor 2 (IGF-2), autoantibodies to insulin, extrapancreatic insulinomas, or glucagon-like peptide (GLP1) [1]. We discuss a case of hepatocellular carcinoma (HCC) producing IGF-2. Although the exact incidence is unknown, it is generally associated with solid tumors of mesenchymal or epithelial origin with HCC being the most common cause of epithelial tumors [2]. Other malignant tumors associated with IGF-2 include leiomyosarcoma, fibrosarcoma, adrenal carcinoma, pheochromocytoma, and renal sarcoma [1]. This rare condition offers several diagnostic challenges due to the limited availability of testing for IGF-1, IGF-2, and pro-IGF-2. However, due to the rising incidence of chronic liver disease and HCC, this case highlights the importance of considering IGF-2 induced hypoglycemia in the differential for a patient presenting with recurrent hypoglycemia in the setting of known malignancy.

## 2. Case Presentation

**Figure 1:**
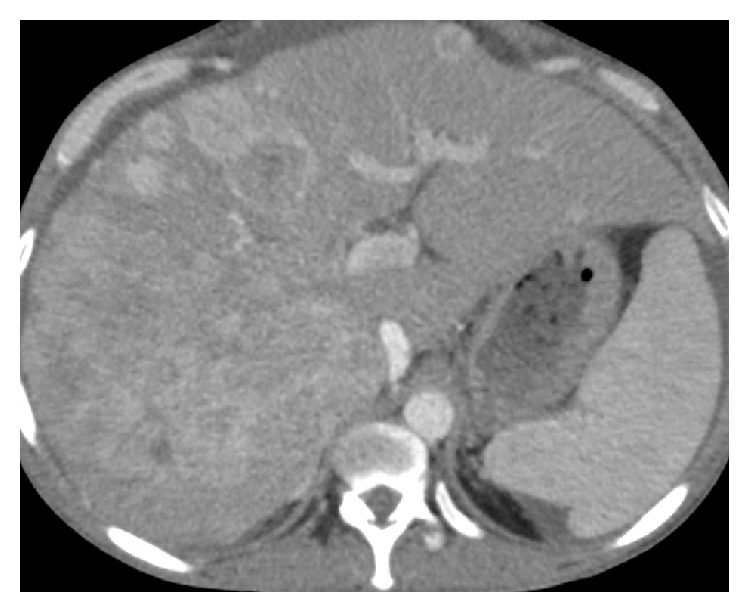
Multiphase abdominal computed tomography: Axial view through the liver showing extensive multifocal HCC.

**Figure 2:**
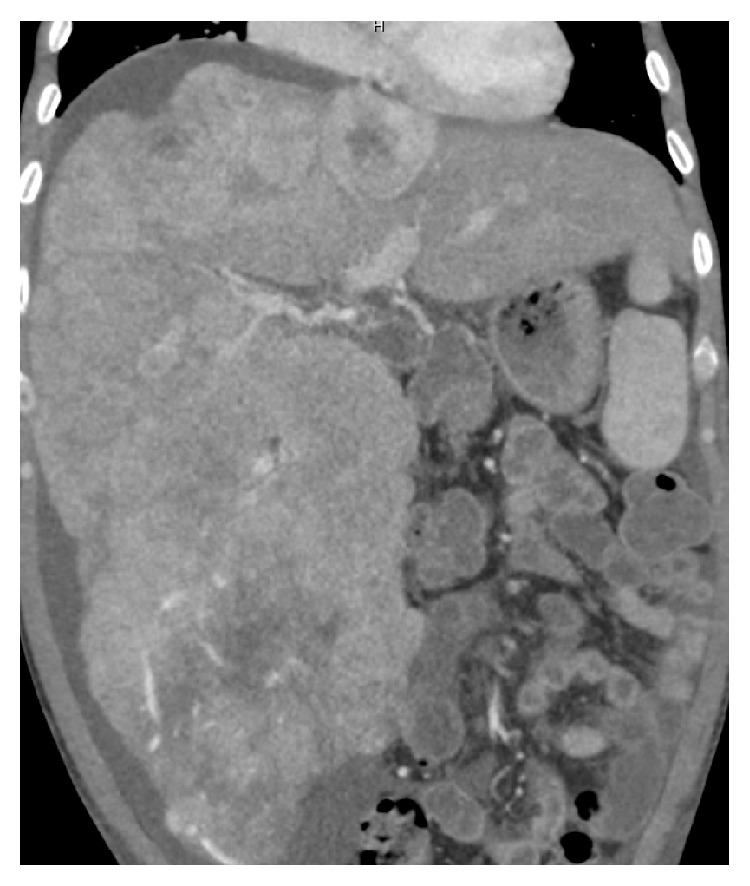
Multiphase abdominal computed tomography: Coronal view through the liver showing extensive multifocal HCC.

We present a 38-year-old man with a history of alcoholic cirrhosis and newly diagnosed hepatocellular carcinoma who presented with sudden onset of abdominal pain. He presented with similar symptoms to another institution 2 months prior, and was diagnosed with HCC. Shortly thereafter, he underwent one round of chemoembolization though the specifics of his prior treatment were unknown. He remained asymptomatic until he presented to our emergency department with sudden onset of right upper quadrant abdominal pain. Past medical and social histories were significant for chronic alcohol use and alcoholic cirrhosis diagnosed several years prior. Upon arrival, a multiphase abdominal computed tomography (CT) scan demonstrated multifocal HCC with local invasion of the right hepatic vein and right portal vein (Figures 1 and 2). CT of the thorax showed no signs of distant metastatic disease. During his hospitalization, he experienced 40–50 episodes of symptomatic and asymptomatic hypoglycemia (38–67 mg/dL) requiring intravenous dextrose administration. There was initial concern for severe hypoglycemia due to prolonged food intolerance in the setting of poor appetite. However, after several episodes of hypoglycemia, further evaluation was pursued. Assessment revealed a normal morning cortisol level of 14.6 mcg/dL (normal 4.8–19.5 mcg/dL) and a mildly elevated thyroid stimulating hormone (TSH) level of 6.04 uIU/mL (normal 0.5–3.5 uIU/mL) with a normal free thyroxine level of 1.65 ng/dL (normal 0.93–1.7 ng/dL). Levels of insulin <0.4 uIU/mL (normal 2.6–24.9 uIU/mL) and C-peptide <0.1 ng/mL (normal 1.1–4.4 ng/mL) measured during an episode of hypoglycemia were low, indicating hypoinsulinemic hypoglycemia. The patient had no history of diabetes mellitus and was not on oral hypoglycemic agents. Two random levels of IGF-2 were elevated at 531 ng/mL and 535 ng/mL (normal 38–267 ng/mL) suggesting a diagnosis of HCC with production of IGF-2.

Hypoglycemia was initially treated with intravenous dextrose infusion. After work-up revealed elevated IGF-2 levels, nasogastric tube with enteral feeds and prednisone 40 mg/day were initiated, and he had no further episodes of hypoglycemia. Given his extensive tumor burden, he was started on immunotherapy with nivolumab. Shortly after completing his first treatment with nivolumab, he opted to pursue home hospice and passed away.

## 3. Discussion

The liver is the principal organ responsible for the production of IGF-1, IGF-2 and its regulating binding proteins, and insulin-like growth factor binding proteins (IGFBP). As demonstrated in murine models, TIH associated with IGF-2 is caused by the overexpression of the IGF-2 gene by the tumor, leading to an elevation of IGF-2 or its precursor pro- or big-IGF-2 [3]. The IGF-2 gene is structurally homologous to insulin and maintains insulin-like activity [1]. In a healthy individual, pro-IGF-2 accounts for 10–20% of the total IGF-2, while it accounts for 60–80% of the IGF-2 in a patient with NICTH [4]. Pro-IGF-2 and IGF-2 suppress both insulin and growth hormone (GH), which in turn decreases the production of IGF-1 and IGFBP-3, the principal binding protein for IGF-2. Furthermore, pro-IGF-2 inherently binds to IGFBP-3 poorly, thereby further increasing the availability of pro-IGF-2 to stimulate insulin receptors and glucose uptake. Continued stimulation of insulin receptors suppresses free fatty acid release and inhibits gluconeogenesis, glycogenolysis, and ketogenesis by the liver, further exacerbating hypoglycemia [1, 2, 4].

**Figure 3:**
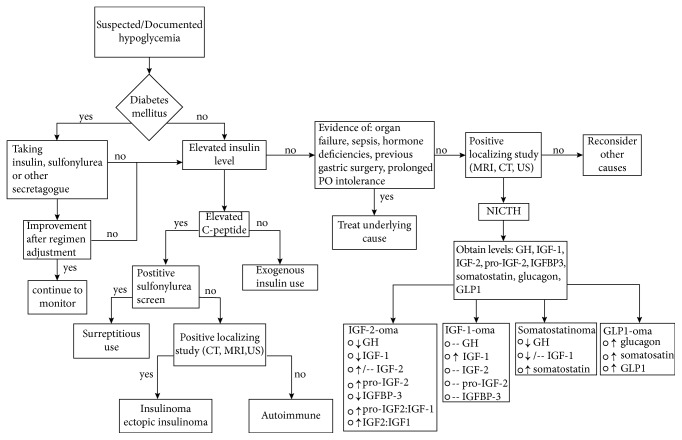
Diagnostic algorithm for patients with hypoglycemia. CT: computed tomography; MRI: magnetic resonance imaging; US: ultrasonography; GH: growth hormone; IGF: insulin-like growth factor; IGFBP: insulin-like growth factor binding protein; GLP: glucagon-like peptide; NICTH: non-islet cell tumor hypoglycemia; -- normal levels.

Evaluation of hypoglycemia will reveal low levels of insulin, pro-insulin, C-peptide, and *β*-hydroxybutyrate. Levels of GH and IGF-1 will also be low due to the suppressive effects of IGF-2. Total IGF-2 may be elevated or normal, while pro-IGF is elevated. The ratio of pro-IGF-2/IGF-2 and IGF-2/IGF-1 is usually elevated 3 : 1, and it may be helpful in cases where levels of IGF-2 are within the normal range (Figure 3). A ratio greater than 10 : 1 of IGF-2/IGF-1 is nearly diagnostic [2, 5, 6].

Initial management of hypoglycemia in NICTH is aimed at achieving and maintaining euglycemia. Supplementation with enteral and/or parenteral nutrition should be initiated promptly to prevent life threatening complications of severe hypoglycemia. Management of an IGF-2 producing tumor with surgical resection can potentially resolve hypoglycemia [2, 7, 8]. For patients with advanced disease, radiation, and/or chemotherapy have been shown to reduce hypoglycemia, although to a lesser degree [7]. When hypoglycemia persists after surgery, radiation, and/or chemotherapy, medical therapy with glucocorticoids or recombinant GH have been utilized. Glucocorticoids act by promoting hepatic gluconeogenesis, promoting lipolysis, and reducing peripheral glucose uptake. Glucocorticoids also help to decrease the levels of pro-IGF-2 either by decreased tumor production or increased clearance. Nevertheless, the effects of glucocorticoids are variable among patients, requiring close titration, and therapy is ineffective when it is discontinued. Recombinant GH functions similarly by increasing gluconeogenesis and glycogenolysis [2, 9].

Although NICTH is a rare cause of TIH, there is a well-established association between mesenchymal and epithelial tumors and production of IGF-2. Given the mortality associated with severe hypoglycemia, there should be a high clinical suspicion for hypoinsulinemic hypoglycemia in patients with recurrent hypoglycemia in the presence of known malignancy.
